# Temperature and Rain Moderate the Effect of Neighborhood Walkability on Walking Time for Seniors in Barcelona

**DOI:** 10.3390/ijerph17010014

**Published:** 2019-12-18

**Authors:** Xavier Delclòs-Alió, Oriol Marquet, Guillem Vich, Jasper Schipperijn, Kai Zhang, Monika Maciejewska, Carme Miralles-Guasch

**Affiliations:** 1 Research Group on Mobility, Transportation and Territory (GEMOTT), Department of Geography, Autonomous University of Barcelona, 08193 Cerdanyola del Vallès, Spain; xavier.delclos@uab.cat (X.D.-A.); guillem.vich@uab.cat (G.V.); .; 2 Barcelona Institute for Global Health (ISGlobal), 08003 Barcelona, Spain; oriol.marquet@isglobal.org; 3 Research Unit for Active Living, Department of Sport Science and Clinical Biomechanics, University of Southern Denmark, 5230 Odense, Denmark; jschipperijn@health.sdu.dk; 4 Department of Epidemiology, Human Genetics and Environmental Sciences, University of Texas School of Public Health, Houston, TX 77030, USA; kai.zhang@uth.tmc.edu; 5 Research Group on Mobility, Transportation and Territory (GEMOTT), Department of Geography & Institute of Environmental Science and Technology (ICTA), Autonomous University of Barcelona, 08193 Cerdanyola del Vallès, Spain; carme.miralles@uab.cat

**Keywords:** physical activity, walking, walkability, weather, seniors, accelerometer, GPS, Barcelona

## Abstract

Walking is the most accessible form for seniors to engage in daily light or moderate physical activity. Walking activity depends on both individual and environmental factors, the latter including how walkable a given setting is. Recent papers have pointed at the relevance of also considering meteorological conditions in relation to the walking behavior of older adults. This paper explores the combined effect of neighborhood walkability, temperature and rain on daily walking time among seniors residing in Barcelona. Daily walking time was extracted from 7-day GPS (Global Positioning System) devices and accelerometer data of 227 seniors residing in the Barcelona Metropolitan Region (Spain). Temperature and rain data were extracted from official governmental weather stations. Mixed-effects linear regression models were adjusted to test the combined association between weather and walkability on daily walking time. Neighborhood walkability is positively associated with walking time among seniors, while rain generally deters it. Additionally, this study demonstrates that temperature and rain modify the effect of residential walkability on senior walking activity: low temperatures are particularly associated with lower walking activity among those residing in low walkable areas, while the presence of rain presents a negative association with walking time in high walkable environments. The combined effect of walkability and weather should be considered both in design actions that aim at improving walking infrastructure and also in prevention programs aimed at encouraging daily walking among seniors.

## 1. Introduction

Maintaining an active lifestyle is a key factor for a healthy aging process and helps ensuring quality of life among older adults [1]. Daily moderate physical activity (PA) engagement is now well established as a measure to reduce the risk of health issues such as coronary heart disease, type 2 diabetes, certain types of cancer, and prevent mental health problems such as depression [2,3]. PA also provides higher levels of independence [4] and can be regarded as a socializing mechanism [5]. However, a large number of seniors in countries such as Spain are still generally sedentary; approximately 40% of the elderly do not meet the World Health Organization’s physical activity recommendations [6,7].

Walking is the most accessible and common strategy to engage in daily light or moderate PA for older adults [8]. However, walking engagement largely depends on individual characteristics as well as on a wide variety of environmental factors [9]. In relation to the latter, it has been shown that the characteristics of the built environment play a significant role in allowing or deterring individuals to walk on a daily basis [10], especially for older adults [11,12]. In this context, the analysis of “walkability” of the urban environment has gained momentum in the past decade. Walkability can be referred to as how friendly a given place is for pedestrians, for instance, in terms of safety or comfort [13], but can also be described more broadly as the capability or the conduciveness of the environment in allowing walking trips on a daily basis [14,15]. The latter, which is the definition of interest for this paper, is commonly analyzed as the combination of land use mix, residential and retail density and urban form [15]. While applicable to the general population, walkability has been proven to be especially relevant for older adults, not only by increasing walking levels [16] but also by preventing falls [17] and in improving social life [5].

However, other environmental factors also have an effect on different forms of outdoor activity. This is the case of meteorological conditions [18,19], but their effect on daily walking activity has been explored to a lesser extent. This gap is especially evident in relation to older adults, even though they are particularly sensitive to weather conditions and extreme weather events [20,21]. In general, previous research relating weather to outdoor PA or walking engagement has provided mixed results. Witham et al. (2014) found that higher temperatures and longer day-light length were associated with higher activity levels among seniors [22]. Similarly, Wu, Luben, Wareham, Griffin and Jones (2017) argued that adverse weather conditions, including high precipitation and low temperatures, were associated with 10% lower average physical activity compared to the best conditions [23]. However, Durand, Zhang, and Salvo (2017) conducted a detailed analysis with a large sample in California, US, using time-matched weather and physical activity related to transport, and found insignificant correlations between activity and weather variables [24].

To the best of our knowledge, few papers have explored the combined effect of walkability and weather conditions specifically for older adults. Ye, Fei, and Mei (2017) evidenced that physical environment characteristics affects walking changes in winter and summer [25]. The previously mentioned study by Witham et al. (2014) also found that the effect of weather in relation to walking was subject to differences in terms of rural and urban environments [22]. Furthermore, and in colder settings, Clarke et al. (2017) found that under snow conditions, older adults who lived in very walkable neighborhoods walked to 25% fewer destinations [26]. Lastly, by analyzing a Mediterranean context, Colom et al. (2019) found that while route walkability had an influence on accelerometer-measured physical activity, rainy conditions during the accelerometer wearing period modified this association [27].

In order to address the research gap identified in relation to the combined effect of walkability and weather conditions in a particularly sensitive group to both variables, this paper presents an analysis of objectively-measured walking activity of seniors residing in a Mediterranean environment, one of the regions that is predicted to be considerably affected by a changing climate in the near future [28]. This paper has two research aims. Firstly, to understand the independent effect of neighborhood walkability, rain and temperature on daily walking time among seniors in a Mediterranean environment. Secondly, to understand if the effect of rain and temperature on walking varies when walkability at the residential environment is considered. To address these questions, we used daily walking time of adults over 65 years old living in the Barcelona Metropolitan Region (Spain) by matching GPS and accelerometer data to daily weather conditions.

## 2. Materials and Methods

### 2.1. Data

**Figure 1:**
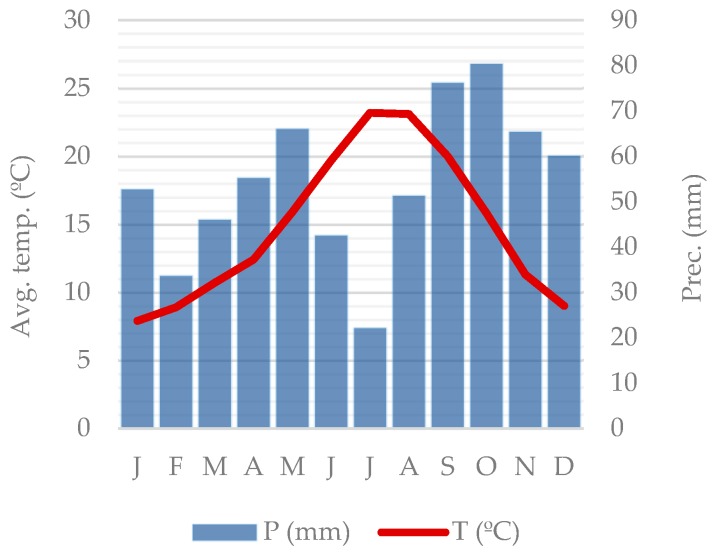
Climograph of the study area. Source: Own production based on weather data for Barcelonès from the reference period 1970–2000. Data were obtained from the Catalan Meteorological Service.

Data in this study were extracted from the project “*Ciudad, calidad de vida y movilidad activa en la tercera edad. Un análisis multimetodológico a través de Tracking Living Labs*” (RecerCaixa 2016) designed to explore the relationship between the built environment and physical activity engagement of older adults in a Mediterranean environment. The study was set in the Barcelona Metropolitan Region (BMR hereafter), one of the main urban areas in Spain and in Southern Europe, with approximately 5 million inhabitants. The BMR presents a hot-summer Mediterranean climate (Csa) according to the Köppen classification. It is characterized by dry summers and mild winters, with the majority of precipitation occurring in spring and fall. The climate of Barcelona, summarized in Figure 1, presents an annual daily average temperature of approximately 15 °C, August being the warmest month (daily average of 23.6 °C) and January being the coldest month (daily average of 8.9 °C). Minimum temperatures are around −5 °C and maximum temperatures around 35 °C. The average annual precipitation is between 600 mm and 650 mm, October being the rainiest month and July being the driest. 

A convenience sample of older adults (65 years old and above) was recruited to wear a GPS device (QStarz BT-Q1000X; Qstarz International Co., Ltd., Taiwan, R.O.C.) and an accelerometer (Actigraph GT3X+; ActiGraph LLC, Pensacola, Florida USA) for a 7-day period with the aim of recording their travel behavior and daily physical activity patterns. The recruitment process consisted in contacting senior day centers across the metropolitan region to find potential participants. A pair of researchers traveled to each of the centers that were interested in taking part in the study and explained the aim of the project, the research protocol and the instructions for the devices to all interested seniors. In addition, a snow-ball sampling technique was implemented to contact other seniors that lived in the area but did not attend the center on a daily basis. As an incentive, participants were offered a report on their own physical activity patterns at the end of the study. Willing participants signed an informed consent form before being given an accelerometer and a GPS device. Participants were asked to answer a short questionnaire about their sociodemographic characteristics, their daily mobility patterns and physical activity habits, as well as about their perception of the neighborhood built environment. The study received the approval of the Ethics Committee on Animal and Human Experimentation at the Universitat Autònoma de Barcelona on 2 February 2017 (CEEAH-3656). Data were collected between 12 June 2017 and 19 June 2018. No data collection was conducted on holidays periods in August 2017 and between 20 December 2017 and 9 January 2018 due to low attendance at senior centers and potential out of the norm behavior among participants, as was reported by the seniors in the centers.

From an initial sample of 269 seniors contacted, only 227 were included in the analysis due to ineligibility (i.e., did not leave residence at least once a day or presented signs of dementia) or insufficient participation in the study. Valid participation was set as presenting at least 4 days with 10 h of wear time [29,30]. The final sample consisted of retired individuals, both women (56%) and men (44%), both under 75 years of age (48%) and over 75 (52%). Participants resided all across the metropolitan region, distributed in low walkable (36%), moderate walkable (32%) and high walkable areas (32%) These categories were based on the walkability index developed by Frank et al. (2010) [15], which is described in the following sub-section.

Weather data were obtained from the official Meteorological Service of Catalonia.

### 2.2. Measures and Analysis

GPS provided the geolocation of participants while accelerometer provided the intensity of PA. Data extracted from the GPS device and the accelerometer were processed and aggregated at 15-s intervals using the Physical Activity Location Measurement System (PALMS), developed by the Centre for Wireless and Population Health Systems, University of California (San Diego, CA, USA) [29]. Combining the information from GPS devices and accelerometers, PALMS was used to detect transportation mode (walking, biking and motorized transportation) and to identify outdoor activity based on the GPS’s Signal-to-Noise-Ratio (SNR). Daily time spent walking for transport is used in this study as the dependent variable.

Daily time spent walking was then related to a set of independent variables. Weather related variables are the following: daily average apparent temperature in Celsius degrees (considering both Heat Index and Wind Chill based on the Spanish Meteorological Agency guidelines) and the occurrence of rain (Yes/No). Weather data were obtained for each day of participation, assigning each participant the data corresponding to the closest station from their home address. Walkability was calculated considering population density, retail floor area ratio, land use mix and intersection density, as defined by Frank et al. (2010) [15], using the following expression:

Neighborhood walkability = [(2 × z-intersection density) + (z-net residential density) + (z-retail floor area ratio) + (z-land use mix)].

Intersection density is weighted by a factor of two in order to acknowledge the strong influence of street connectivity on walking activity [15]. Walkability was calculated at a 600 m network buffer around the home address, considering that this threshold approximately corresponds to a 10 min walk from home, which is regarded as an adequate definition of the neighborhood [31,32] These calculations were conducted using ArcGIS 10.5 (ESRI, Redlands, CA)

Several variables were selected from the self-report survey conducted prior to participation in order to serve as co-variates. These included sex, age, access to vehicle and usual transportation mode. Usual transportation mode was a single choice question reflecting the most commonly used mode on a given day. Other variables in the questionnaire were not included in the analysis.

To examine associations between weather variables and walkability with daily walking levels, we first conducted descriptive statistics followed by multilevel linear mixed effects models with user identification number as the random effect, as conducted in similar studies [33]. First, we applied non-parametric tests (the Mann–Whitney U test for binary variables and the Kruskal–Wallis test for categorical variables with more than three categories) to evaluate the association between each independent variable and the outcome, daily walking minutes. Then, we adjusted two different mixed effects regression models with the log-transformed daily amount of walking minutes as the dependent variable. The first model includes the above-mentioned independent variables as explanatory factors. The second model includes a set of two-way interaction terms combining each weather variable with the low and high walkability levels. Lastly, we present the second regression model’s post-estimated values considering all independent variables in order to compare behavior in different walkability settings under diverse weather conditions.

## 3. Results

Table 1 describes the study sample and presents the median values of daily walking minutes considering personal and weather-related variables. Participants in this experiment registered a median of 14.8 min of daily walking time. Men, those under 75 years of age, those with access to vehicle, those with walking as the main mode of transportation and those living in high walkable areas, spent significantly more time walking than other seniors. Regarding weather-related variables, the higher median values of walking time was registered during summer months, which is related to the fact that days with an average apparent temperature over 25 °C present the highest median of walking minutes. Lastly, participants walked significantly less on rainy days.

However, results changed when the relationship between these explanatory factors and daily walking minutes was analyzed jointly in the mixed-effects regression models. Model 1 (Table 2) presents the effect of these independent variables on the log-transformed walking minutes as the dependent variable. Among key independent variables, it is observed that walkability presents a significant positive relationship with daily walking minutes (b = 0.03, *p* < 0.01). While temperature does not seem to present a statistically significant effect in general terms, the presence of rain does present a negative association with daily walking time (b = −0.12, *p* < 0.01). The effect of control variables is the following: men walk significantly more than women (b = 0.28, *p* < 0.01) and older seniors walk less than younger seniors (b = −0.02, *p* < 0.01).

The purpose of Model 2 (Table 3) is to test if weather conditions affect seniors differently in their daily walking activity based on neighborhood walkability levels. Walkability as a continuous variable has been excluded and a set of interaction terms between low and high walkability levels and Temperature and Rain categories were created. The results show that low temperatures (<10 °C) present a significant negative effect, specifically for those residing in low walkable areas (b = −0.15, *p*-value = 0.01). On the other hand, for those residing in high walkable areas, the results suggest that the presence of rain is negatively associated with daily walking minutes (b = −0.12, *p*-value < 0.01). As for control variables, sex and age maintain the effects observed in Model 1, and walking as the main mode of transportation presents a positive association with daily walking time (b = 0.11, *p*-value = 0.05).

According to Model 2 coefficients, we obtain the predicted log-transformed daily walking minutes of each type of participant included in the analysis according to walkability levels at the residential context and different temperature and rain conditions (Table 4).

Considering the combined effect of all the independent variables, it is estimated that seniors residing in high walkable neighborhoods walk 43.47% more time than their counterparts in low walkability areas. However, this difference in walking time based on neighborhood walkability varies when weather is considered. In low temperature conditions (≤10 °C), the difference in walking time between low and high walkable areas considerably increases (129.06%). Considering the results in Model 2, this is due to a significantly lower value observed in low walkability areas (9.30 min). In higher temperatures (>25 °C) those residing in high walkable neighborhoods still walk more time than those in low walkability neighborhoods (36.78%), but the difference between the two groups was reduced. Lastly, while the presence of rain implies lower walking time for all participants in general, the negative effect of rain seems to be particularly relevant for those residing in high walkable environments, which results in a smaller difference between the two groups (32.93%) compared to other residential settings.

## 4. Discussion

This paper explored the combined effects of neighborhood walkability and weather conditions in daily walking time among seniors in the Barcelona Metropolitan Region, Spain, adjusting for personal characteristics. While walking was associated with walkability in the residential context, and rain was associated with lower levels of walking activity, our results suggest that the effect of walkability is moderated by temperature and rain.

First of all and in terms of control variables, the results show that male participants tend to walk more on a daily basis than women, which is in line with previous research, especially considering walking for leisure [34].

In terms of key independent variables, walkability in the residential environment defined as the combination of density, land use mix and number of intersections has once again proven to play a significant role in encouraging walking among older adults, in accordance with previous research [16,35]. In this case, the difference between living in low and high walkable environments suggests an approximate 14% increase in walking time for seniors.

In relation to weather conditions, temperature did not appear as a significant factor in walking levels for the entire sample, even though seniors are generally vulnerable to high temperatures [36]. This result is in line with previous research in other regions of the world that found no significant association between temperature and PA related to transport [24]. In the context of this study, this result could also have been influenced by the fact that no extreme temperatures were registered during the study period, which, in the case of Barcelona, is normal considering the moderate nature of the Mediterranean climate and also strengthened by the modulating effect of the sea. On the other hand, Mediterranean climates are defined by a small amount of days with rain, which helps in understanding the negative association between the presences of rain and lower walking activity among this group of seniors.

Nevertheless, these relationships substantially differ when we focused on the two extreme categories of residential walkability. First, the results suggest that seniors residing in low walkable areas were less likely to walk in low temperature conditions. This could be partially explained by evidence found in previous research, which suggests that walking among older adults residing in car-oriented environments decreases with lower temperatures, and especially under snowy conditions [26]. However, this may not be completely transferrable to this study site due to the lack of snow during the analyzed period. In this case, the decrease in walking in low temperature conditions could be explained by the low availability of destinations and the consequent need to walk farther to reach them, especially for older adults [35], which could mean that these trips could be more sensitive to temperature changes. Moreover, it has to be considered that low walkability areas usually register the lowest temperatures, considering that high walkability settings correspond to core urban centers, which are affected by the urban heat island effect [37].

On the other hand, while temperature does not seem to be playing a specific role among those who reside in high walkable environments, the presence of rain was significantly associated with lower walking levels among this second group of seniors. This is in line with Colom, Ruiz, Wärnberg, et al. (2019), who found that rain especially had an effect on walkable routes in promoting outdoor physical activity among older adults [27]. These results could be explained, on the one hand, by the fact that sidewalks and pedestrian crossings (which are more present compared to low-walkable neighborhoods) are especially slippery and might present puddles in rain conditions and therefore, fear of falling among seniors could be especially aggravated [38,39]. Similarly, walking among seniors in high walkable environments is associated with the presence of benches and places to rest, which are not usable under the rain [38]. Lastly, it should be considered that in urban environments, older adults can feel especially insecure due to the presence of motorized and non-motorized vehicles and the consequent fear of being hit [40]. This feeling of unsafety might be increased under rain and low visibility conditions, since it has been largely evidenced that rain is significantly associated with accidents involving vehicles and pedestrians [41].

This paper has several strengths. First of all, and to the best of the authors’ knowledge, it is one of the first studies to deal with the combined effect of walkability and weather conditions on walking among seniors. In this case, by considering both rain and temperature. Secondly, the analysis was based on objectively measured walking activity measured by combining GPS and accelerometer data, as opposed to self-reported accounts of PA. This provided multiday data and helped in avoiding recall and perception biases.

However, this study is not exempt of limitations that should be taken into consideration. First, considering that these results are based on voluntary participants, the sample might have been biased towards seniors that are generally more active than the average. Second, and regarding GPS data, even though it is more reliable than self-reported measures of walking, it is not exempt of spatial errors, especially in dense urban areas [42]. Third, it also has to be considered that no data were registered during vacation periods in August and two weeks in December and January, which may have had an effect in temperature variability. In terms of weather variables, this analysis is focused on the daily average of temperature and daily occurrence of rain. Future studies could further explore the effect of weather on walking activity by using hourly data during daytime hours in order to have a more precise spatiotemporal match between temperature and rain and walking events, which are more likely to take place during the day. Lastly, the association of walking time with rain could be further analyzed by not only considering the occurrence but also the amount of precipitation.

## 5. Conclusions

This paper dealt with the combined effect of walkability, temperature and rain on walking time among seniors in a Mediterranean context. This is relevant considering that in the context of a changing climate, Mediterranean areas are likely to experience more frequent extreme weather events, specifically by presenting more extreme precipitation occurrences and higher temperatures. Moreover, these changes might be especially relevant for one of the demographic groups more sensitive to weather conditions, older adults. These results imply that when promoting walking as a relatively accessible means to achieve PA recommendation levels among older adults, it is important not only to consider individual physical and mental attributes, but also environmental factors that are likely to play an important role. The results of the study suggest that while walkability is a relevant factor allowing older adults to walk on a daily basis, its effects might be nuanced by weather conditions. While policies that care about promoting PA levels among seniors should keep on improving walkability in the long term, in the short term, those that are focused on car-dependent and low walkable environments could reinforce other forms of PA and socialization during cold months, for instance by reinforcing indoor activities at public or community centers. This would be a similar approach to that suggested for children and schools [43]. On the other hand, and considering the importance of paved surfaces for seniors walking activity [33], efforts aimed at guaranteeing certain levels of walking activity among seniors in urban and high walkable environments should be focusing on safety issues. This would be particularly relevant under rainy conditions, for instance, by investing in street design elements such as sidewalks or curb ramps, that are relevant in seniors’ safety [39,44,45], and therefore contributing to reducing the risk of falling and the risk of accidents under such circumstances.

## Figures and Tables

**Table 1:** Daily walking time in relation to personal characteristics and weather-related explanatory factors.

Explanatory Factor	Days with Data *n* (%)	Walking Minutes ^a^	IQR ^b^	*p* ^c^
Total	1502 (100)	14.8	41.0	
Sex				<0.01
Female	833 (55.5)	9.8	28.5	
Male	669 (44.5)	27.0	57.9	
Age				<0.01
65–75 y. o.	721 (48.0)	23.3	50.8	
≥75 y. o.	781 (52.0)	10.0	29.3	
Access to vehicle				<0.01
Yes	955 (63.6)	18.3	44.5	
No	534 (35.6)	11.0	35.8	
n. d.	13 (0.8)			
Usual transport mode				<0.01
Walking	962 (64.0)	19.3	45.0	
Public transportation	244 (16.2)	8.8	37.4	
Private transportation	282 (18.8)	9.3	29.5	
n. d.	14 (1.0)			
Neighborhood walkability				<0.01
Low	555 (37.0)	10.0	31.5	
Moderate	469 (31.2)	19.0	46.0	
High	478 (31.8)	19.6	49.7	
Season				<0.05
Winter	458 (30.5)	15.0	33.4	
Spring	575 (38.3)	13.0	37.3	
Summer	151 (10.1)	26.8	60.0	
Autumn	318 (21.2)	12.6	45.7	
Apparent temperature ^d^		14.8		<0.01
Less than 10 °C	391 (26.0)	11.8	33.0	
From 10 °C to 25 °C	1046 (69.6)	15.5	41.5	
25 °C or more	65 (4.3)	47.8	78.5	
Rain				<0.01
No	1061 (70.6)	18.8	46.3	
Yes	441 (29.4)	9.8	29.1	

^a^ Median values. ^b^ Interquartile range (IQR). ^c^ Statistical significance (*p*-value) from Non-parametric Mann–Whitney U test (for two-category variables) and Kruskal–Wallis test (for variables with 3 or more categories). ^d^ Following the Spanish Meteorological Agency’s guidelines, temperatures were adjusted by Heat Index for temperatures above 26 °C and 40% of air humidity and Wind Chill effect for temperatures below 10 °C and more than 5 km/h of wind; Heat Index (HI) = −8.78469476 + 1.61139411 × T + 2.338548839 × HR − 0.14611605 × T × HR − 0.012308094 × T^2^ − 0.016424828 × RH^2^ + 0.002211732 × T^2^ × R + 0.00072546 × T × RH^2^ − 0.000003582 × T^2^ × RH^2^; Wind Chill (WC) = 13.1267 + 0.6215 × T − 11.37 × W^0.16^ + 0.3965 × T × W^0.16^; where T is temperature in Celsius, RH is relative humidity in %, and W is wind speed in km/h.

**Table 2:** Model 1: Mixed-effects linear regression model relating daily walking minutes (log-transformed) with personal and weather related independent variables.

**Fixed Effects**	**B**	**Std. Err**	**t**	**p**	**CI (95%)**	
Intersection	2.65	0.31	8.68	<0.01	2.05	3.25
Sex (Female = Ref.)	0.28	0.06	5.07	<0.01	0.17	0.39
Age	−0.02	0.00	−5.21	<0.01	−0.03	−0.01
Neighborhood Walkability Index	0.03	0.01	2.65	<0.01	0.01	0.05
Access to vehicle (No = Ref.)	−0.11	0.06	−1.68	0.094	−0.24	0.02
Usual transportation mode (Not walking = Ref.)	0.08	0.06	1.41	0.159	−0.03	0.20
Temperature less than 10 °C (No = Ref.)	−0.05	0.04	−1.18	0.237	−0.13	0.03
Temperature 25 °C or more (No = Ref.)	0.12	0.10	1.18	0.240	−0.08	0.32
Rain (No = Ref.)	−0.12	0.03	−3.80	<0.01	−0.18	−0.06
**Random Effects**	**B**	**Std. Err**	**Wald Z**	**p**	**CI (95%)**	
Residual	0.16	0.01	21.62	<0.01	0.15	0.17
Subjects	0.11	0.01	7.59	<0.01	0.09	0.15

B = Coefficient estimate; Std. error = Standard error; *p* = *p*-value; CI = Confidence interval. Intraclass coefficient (ICC): 0.538 (null model), 0.415 (full model). Portion of individual variance explained by fixed effects: 0.397.

**Table 3:** Model 2: Mixed-effects linear regression model relating daily walking minutes (log-transformed) with personal independent variables and interaction terms between walkability levels and weather conditions.

**Fixed Effects**	**B**	**Std. Err**	**t**	**p**	**CI (95%)**	
Intersection	2.67	0.30	8.83	<0.01	2.07	3.26
Sex (Female = Ref.)	0.32	0.05	5.86	<0.01	0.21	0.42
Age	−0.02	0.00	−5.99	<0.01	−0.03	−0.02
Access to vehicle (No = Ref.)	−0.05	0.06	−0.88	0.378	−0.17	0.07
Usual transportation mode (Not walking = Ref.)	0.11	0.06	2.00	<0.05	0.00	0.23
Low walkability × Temperature below 10 °C	−0.15	0.06	−2.48	<0.05	−0.27	−0.03
Low walkability × Temperature 25 °C or more	0.16	0.14	1.22	0.224	−0.10	0.43
Low walkability × Precipitation (Yes)	−0.06	0.05	−1.26	0.207	−0.16	0.04
High walkability × Temperature below 10 °C	0.00	0.07	0.02	0.987	−0.13	0.13
High walkability × Temperature 25 °C or more	0.15	0.15	1.00	0.317	−0.14	0.45
High walkability × Precipitation (Yes)	−0.12	0.05	−2.61	<0.01	−0.21	−0.03
**Random Effects**	**B**	**Std. Err**	**Wald Z**	**p**	**CI (95%)**	
Residual	0.16	0.01	21.82	<0.01	0.15	0.18
Subjects	0.11	0.01	7.64	<0.01	0.09	0.15

B = Coefficient estimate; Std. error = Standard error; *p* = *p*-value; CI = Confidence interval. Intraclass coefficient (ICC): 0.538 (null model), 0.4105 (full model). Portion of individual variance explained by fixed effects: 0.399.

**Table 4:** Predicted ^a^ walking minutes ^b^ considering neighborhood walkability, temperature levels and rain.

	Total	Temperature		Rain	
		≤10 °C	>25 °C	No	Yes
Low walkability	14.87	9.30	41.08	15.99	12.69
High walkability	21.34	21.30	56.19	24.29	16.87
Diff. (min.)	6.47	12.00	15.11	8.30	4.18
Diff. (%)	43.47	129.06	36.78	51.90	32.93

^a^ Adjusted by variables included in Model 2.; ^b^ Back-transformed predicted values.
